# Postprandial thermogenesis and respiratory quotient in response to galactose: comparison with glucose and fructose in healthy young adults

**DOI:** 10.1017/jns.2015.41

**Published:** 2016-01-29

**Authors:** Nathalie Charrière, Jean-Pierre Montani, Abdul G. Dulloo

**Affiliations:** Department of Medicine, Division of Physiology, University of Fribourg, CH 1700 Fribourg, Switzerland

**Keywords:** Energy expenditure, Respiratory quotient, Thermogenesis, Sugars, Galactose, Fructose, REE, resting energy expenditure, RQ, respiratory quotient

## Abstract

Circumstantial evidence suggests that substitution of glucose or sucrose by the low-glycaemic index sugar galactose in the diet may lead to greater thermogenesis and/or fat oxidation. Using ventilated hood indirect calorimetry, we investigated, in twelve overnight-fasted adults, the resting energy expenditure (REE) and respiratory quotient (RQ) for 30 min before and 150 min after ingestion of 500 ml of water containing 60 g of glucose, fructose or galactose in a randomised cross-over design. REE increased similarly with all three sugars, reaching peak values after 50–60 min, but its subsequent fall towards baseline values was faster with galactose and glucose than with fructose (*P* < 0·001). RQ increased with all three sugars, but to a much greater extent with galactose and fructose than with glucose, particularly after 1 h post-ingestion. When ingested as a sugary drink, postprandial thermogenesis and utilisation of fat after galactose are not higher than after glucose or fructose.

The consumption of refined sugars in food and beverages has risen sharply in recent decades and is thought to contribute importantly to the current epidemic of obesity and cardiometabolic diseases^(^^1^^)^. Most of the added sugars are consumed as the disaccharide sucrose or as its monosaccharide moieties glucose and fructose. Although fructose, by virtue of its low postprandial glucose and insulin responses (i.e. low glycaemic index) and greater postprandial thermogenesis than glucose, was initially viewed as being advisable for diabetic patients and beneficial for weight control, it is nowadays regarded as the more harmful sugar component^(^^2^^)^. Indeed, chronic high consumption of fructose in substitution for glucose in the diet has been shown to lead to a more adverse lipid profile and greater risks for central obesity, diabetes and CVD^(^^2^^,^^3^^)^.

While comparison between these two dietary monosaccharides – glucose and fructose – continues to be a major focus of research about their differences in adverse health effects, there is increasing interest in the potentially beneficial effects of another dietary monosaccharide – the milk sugar galactose – which in combination with glucose constitutes the disaccharide lactose. As most galactose undergoes conversion in the liver, its release as glucose into the blood is delayed and it could thus provide an energy source of choice, with low glycaemic index and a low insulinaemic response^(^^4^^,^^5^^)^, while being absorbed at the same rate as glucose^(^^6^^)^. Moreover, galactose ingestion in humans has recently been associated with a decrease in hunger sensation^(^^7^^,^^8^^)^, and an increase in fat mobilisation and oxidation^(^^9^^)^, which would be in line with its much lesser impact on circulating insulin than glucose^(^^4^^,^^5^^)^. These findings have led some authors^(^^9^^)^ to put forward the hypothesis that weight loss may be facilitated when a moderate energy restriction is combined with galactose consumption, and that diets with galactose as a source of carbohydrates could be useful in the management of obesity and type 2 diabetes.

Further evidence in support of a greater stimulatory effect of galactose on fat oxidation, and possibly energy expenditure, may also be derived from studies in the rat showing that (i) the addition of lactose to a high-fat diet resulted in less body fat accumulation despite no difference in energy intake^(^^10^^)^, and (ii) a lower rate of weight gain, associated with increased sympathetic nervous system activity in abdominal adipose tissue after chronic high galactose feeding when compared with high glucose or fructose isoenergetic feeding^(^^11^^)^.

The above-mentioned findings in human subjects and animals raise the question of whether the thermic effect of galactose might also be different from that of the other two dietary monosaccharides. In this context, an early study in healthy adults reported no significant difference between galactose and glucose on postprandial resting energy expenditure (REE)^(^^12^^)^. However, given that in the latter study large amounts of sugars were ingested as single boluses (140 g in men and 110 g in women), but postprandial REE measurements were made over a time period not long enough to capture most of the thermic effect of such large amounts of sugars, the question of whether the thermic effect of galactose is different or not from that of glucose remains unanswered. Furthermore, no study has directly compared the effects of galactose and fructose on postprandial REE and respiratory quotient (RQ).

The aim of our study therefore was to investigate potential differences in postprandial thermogenesis and RQ between galactose, glucose and fructose in a cross-over study design in young adults.

## Materials and methods

### Subjects

A total of twelve healthy young non-obese adults (six men and six women) were studied; their mean age and physical characteristics are shown in Table 1. Smokers, pregnant women, individuals taking medication and those reporting any metabolic disease or lactose and fructose intolerance were excluded. The present study was conducted according to the guidelines laid down in the Declaration of Helsinki, and all procedures involving human subjects were approved by the Cantonal Ethics Committee. Written consent was obtained from all subjects.

**Table 1.** Characteristics of subjects (*n* 12) (Mean values with their standard errors, and ranges)

	Mean	sem	Range
Age (years)	24	0·5	20–27
Weight (kg)	69·1	4·4	51·7–96·5
Height (cm)	172·0	3·3	155·5–188·2
BMI (kg/m^2^)	23·1	0·8	20·0–29·5
Fat mass (kg)	13·7	1·7	6·6–23·1
Fat-free mass (kg)	55·4	4·0	39·4–76·5
Total fat (%)	20·1	2·3	9·2–36·9
Abdominal fat (%)	23·4	2·5	9·9–38·7

### Experimental design

All participants were requested to avoid physical activity, caffeine and dietary supplements in the 24 h prior to testing, and to use motorised transport to reach the laboratory. Anthropometry and body composition (using bioimpedance analysis) were measured as previously described^(^^13^^)^. The metabolic measurements were conducted in the morning after an overnight fast, with the participant seated comfortably in a car seat adapted for continuous measurements of REE and RQ by ventilated hood indirect calorimetry (Cosmed Quark RMR; Cosmed srl), as previously described^(^^13^^)^. The O_2_ analyser (with paramagnetic O_2_ sensor) and CO_2_ analyser (with an IR digital sensor) offer a fast response time to measure O_2_ and CO_2_ changes within 120 ms; the range of O_2_ measurement is from 0 to 30 % and that of CO_2_ measurement from 0 to 10 %, both with an accuracy of ±0·02 %. Prior to each test, the gas analysers were calibrated using a certified gas mix (5 % CO_2_, 16 % O_2_ and 79 % N_2_). The turbine flowmeter has an accuracy of ±2 % and was calibrated prior to each test with a 3 litre syringe. Flow rate during each test was fixed between 30 and 37 litres/min so as to maintain the concentration of CO_2_ in air flowing out of the canopy between 0·7 and 1·0 %. In order to avoid baseline drift the Cosmed Quark RMR system automatically recalibrates every 5 min (over a 55 s period). REE was calculated according to the Weir equation^(^^14^^)^, and RQ was calculated as the ratio of CO_2_ produced to O_2_ consumed (i.e. RQ = VCO_2_/VO_2_). As short-term urinary collections to assess total N excretion may not be representative of the protein oxidised during the measurement itself, they were not obtained in this study, and assumed to be 13 g/24 h; the latter value reflects urinary N excretion of subjects in the post-absorptive (fasted) state^(^^15^^)^. It should be noted that this assumption will not significantly influence the relative partition between carbohydrate and fat oxidation determined by indirect calorimetry because (i) the average overnight-fasted RQ of the subjects (about 0·80) is close to the RQ of protein (about 0·82), (ii) the proportion of total oxidation derived from protein is small (12–15 %) and (iii) in response to carbohydrate ingestion (as in our study here), this proportion is likely to be even smaller due to the potential protein-sparing effect of carbohydrate on protein oxidation.

On each test day, upon arrival in the laboratory at approximately 08·00 hours, the subject rested in the seated position for 15–20 min. This was followed by 30–40 min of baseline measurement, during which stabilisation of REE was achieved. Stabilisation was defined as no more than 2 % variability of REE, with no consistent upward or downward trend. The subject then drank, in 4 min, a 500 ml beverage containing distilled water, 10 ml of lemon juice and 60 g of d(+)glucose, d(+)galactose or d(−)fructose (Argos Organics, Chemie Brunschwig SA) in a randomised cross-over design; lemon juice was added in order to mask differences in taste of the different sugars. The ventilated hood was then replaced and post-drink metabolic monitoring continued for a further 150 min. In order to reduce boredom and accompanying stress, the participants were permitted to watch a calm movie or a documentary. All participants were blinded as to the order in which they received the sugar drinks.

### Data and statistical analysis

The required number of subjects (*n* 12) was determined by the application of power analysis to detect a higher thermic effect (calculated as percentage of ingested energy) of galactose or fructose (9·4 %) than that for glucose (6·6 %)^(^^16^^)^, with a standard deviation of 3·3 % for the population^(^^17^^)^, and based upon a desired statistical power of 80 % and a 5 % significance level. All data are presented as means with their standard errors. Statistical analysis was performed by ANOVA for repeated measures, with time and drink as within-subject factors using the statistical software Statistix version 8.0 (Analytical Software), and applied to baseline (pre-drink) data, as well as across pre-drink and post-drink data. The thermic response to each sugar drink was calculated as the integrated change in post-drink REE (assessed as the AUC by the trapezoidal rule) expressed as a percentage of energy intake, and compared using one-way repeated-measures ANOVA followed by Tukey's test for multiple pair-wise comparisons. The level of statistical significance was set as *P* < 0·05. Linear regressions and correlations of postprandial changes in REE and RQ against body weight and body composition were performed using the computer software Prism (version 5.02; GraphPad Software Inc.).

## Results and discussion

No significant differences were found in baseline (pre-drink) REE or RQ values across days: the mean baseline REE values for glucose, galactose and fructose were 4·27 (se 0·30), 4·45 (se 0·33) and 4·43 (se 0·34) kJ/min, respectively; the mean baseline RQ values for glucose, galactose and fructose were 0·81 (se 0·02), 0·79 (se 0·02) and 0·78 (se 0·02), respectively.

**Fig. 1.**
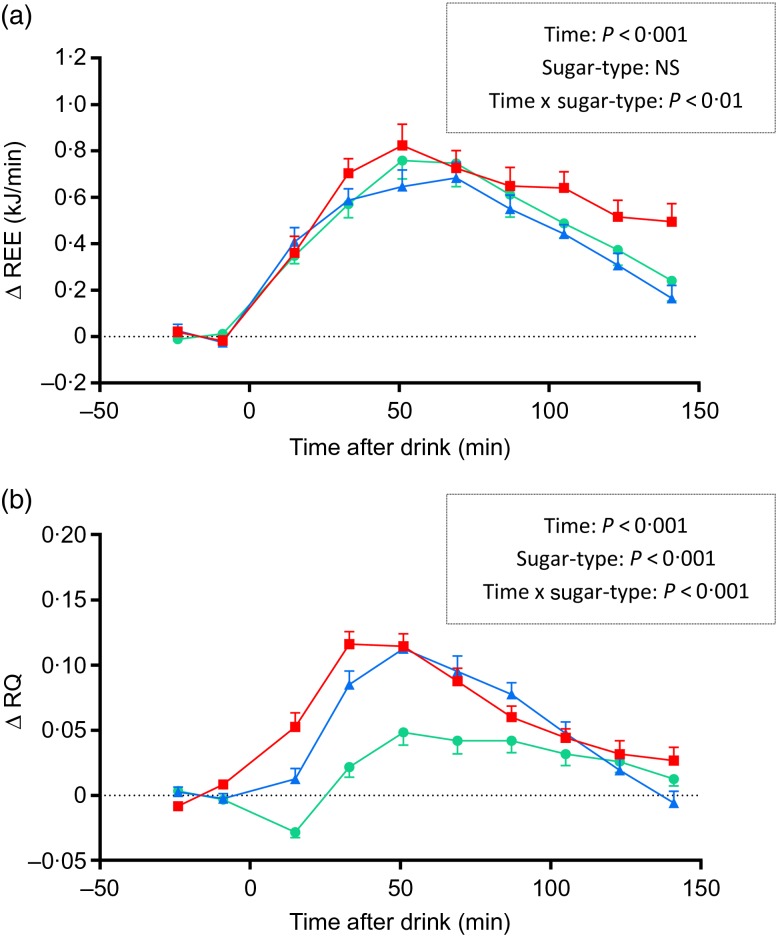
Time course of changes in (a) resting energy expenditure (Δ REE) and in (b) respiratory quotient (Δ RQ) after drinks containing glucose (–●–), galactose (–▲–) and fructose (–■–). Values are means, with standard errors represented by vertical bars. The results of repeated-measures ANOVA assessing statistical differences for the effects of time, sugar-type, and sugar-type × time interaction are provided in the rectangular boxes.

In response to ingestion of the sugary drinks, REE increased significantly above baseline. The temporal increases in REE during the first 1 h were similar for all sugars (Fig. 1(a)), with peak values being reached between 50 and 60 min post-drink (∆ = 0·76 kJ/min for glucose, 0·65 kJ/min for galactose and 0·82 kJ/min for fructose; all *P* < 0·001 relative to baseline). The subsequent fall in REE towards baseline values was, however, slower with fructose than with glucose or galactose: the REE during the last 1 h (i.e. between 90 and 150 min) was significantly higher for fructose than for glucose or galactose; ANOVA indicated a significant interaction between sugar-type and time (*P* < 0·01). Unlike for fructose, the thermic response to glucose and galactose was more than 90 % completed by 150 min post-drink, and this is reflected in the lower thermic effect of glucose (6·9 %) and galactose (6·3 %) than for fructose (8·1 %).

The results on RQ, presented in Fig. 1(b), indicate that RQ increased following the ingestion of all sugars, reaching peak values within 45–60 min before declining to reach baseline values by 150 min post-drink. However, the changes in RQ were faster and greater with fructose and galactose than with glucose during the first 1 h post-drink (0·08–0·09 *v*. 0·02, *P* < 0·001), leading to an overall strong effect of sugar-type (*P* < 0·001) as well as a sugar-type x time interaction (*P* < 0·001). Further analysis of the data which included sex as a covariate in the ANOVA model indicated that the differential REE or RQ responses to the three monosaccharides were observed independently of sex (data not shown).

Thus, in the present study comparing postprandial REE and RQ responses to all three dietary monosaccharides in a cross-over design, it is found that while the increases in REE and RQ after fructose are both greater than after glucose, the effect of galactose ingestion on postprandial REE is similar to that of glucose (and hence lesser than that of fructose), whereas its impact on RQ is similar to that of fructose, and hence higher than that of glucose.

With respect to the greater REE and RQ effects of fructose compared with glucose ingestion, our results are in line with those of previous studies comparing higher amounts (75–100 g) of these two sugars^(^^16^^)^. We chose a dose of 60 g of sugar for our test drinks for two reasons. First, in our experience, the ingestion of greater amounts of fructose (>60 g) increases the risks of fructose malabsorption leading to gastrointestinal problems. Second, we estimated that the ingestion of 60 g (i.e. 240 kcal; 1004 kJ) of our reference sugar, glucose, would produce a thermic response profile that would be completed or near-completed within 150 min. Indeed, as our study here indicates, the thermic effect of glucose, as that for galactose, was >90 % completed within this time period lasting 150 min. Furthermore, the post-drink REE response profile of galactose is found to be similar to that of glucose in the ascending phase, across the plateau phase and in the recovery towards baseline values.

Our results also confirm past observations of differential RQ responses to glucose and fructose^(^^3^^,^^16^^,^^17^^)^, with fructose ingestion having repeatedly been shown to lead to a greater increase in RQ than glucose, namely +0·06 over 120 min; this compares well with a greater increase in RQ of +0·05 over 150 min after fructose than glucose in our study. Furthermore, it was shown that the kinetics of RQ across the postprandial measurement period are similar for galactose and fructose, and that for all three sugars, the RQ values have returned to baseline levels by 150 min post-drink.

We chose to give a fixed sugar load (60 g of each test sugar) to all our participants despite body-weight variations both between- and within-sex because we found no evidence in the literature for a relationship between body weight and the thermic response to a given food or specific macronutrient, at least in young adult subjects who are healthy and non-obese. Using our data presented here, we also checked for a potential correlation between the thermic effect of the sugar drinks and body weight but found none – which further underscores the lack of rationale for a weight-adjusted sugar load administration. Furthermore, we found no correlation between the increase in REE or in RQ in response to any of the monosaccharides with body weight, fat-free mass, total body fat or abdominal fat.

It is well known that these three monosaccharides differ in their absorption rates and/or metabolic fates, thereby resulting in their different rates of appearance in (and clearance from) the circulation, and in different concentrations across time in the systemic circulation. Following oral ingestion, fructose, which is absorbed at a rate which is considerably slower than that of glucose and galactose, is largely taken up by the liver and rapidly phosphorylated to fructose-1-phosphate, and further metabolised to produce 3-carbon intermediates in the glycolytic pathway^(^^18^^)^. In humans, about 40–50 % of oral fructose is converted to glucose by the liver, with a minor part (up to 15 %) stored as glycogen and most released into the systemic circulation with 4–6 h post-ingestion^(^^18^^)^. Although galactose is absorbed by the same active transport system and at the same rate as glucose^(^^6^^)^, the fact that most galactose, like fructose, is converted in the liver, means that their release as glucose into the blood is delayed, with resultant low glycaemic index and a low insulinaemic response^(^^4^^,^^5^^)^. Thus, galactose and fructose, unlike glucose, require metabolic transformation by the liver into other substrates (glucose, lactate or fatty acids) that are readily metabolised by extrahepatic cells. By contrast, most of the ingested glucose escapes the splanchnic bed raising plasma insulin which will inhibit hepatic glucose production from glycogen, increase plasma lactate, and influence glucose clearance in peripheral tissues, particularly in skeletal muscle. The potential significance of some of these differences in the absorption rate and metabolic fates of the three monosaccharides are discussed below pertaining to our findings here regarding their different impact on whole-body REE and RQ.

First, the greater thermic effect of fructose than glucose could, to a large extent, be attributed to the extra ATP requirements linked to conversion of fructose to glucose in the liver, and a minor part possibly to the energy cost of *de novo* fatty acid synthesis from fructose which is known to be more potent than glucose as a stimulator of *de novo* lipogenic enzymes^(^^16^^)^. This latter pathway may also contribute to the higher RQ observed with fructose than with glucose. Indeed, the fact that the ingestion of both galactose and fructose in our study here resulted in equally greater increases in RQ than after glucose, but that a greater increase in REE compared with glucose is only observed after fructose but not galactose ingestion, raises the question as to whether the increased RQ with galactose resides solely in increased carbohydrate oxidation, without entering the energetically costly *de novo* fatty acid synthesis pathway. Nonetheless, based upon evidence from *in vitro* studies in rat adipocytes suggesting that galactose can promote glucose utilisation for fatty acid synthesis^(^^19^^)^, the issue of whether galactose is a less potent stimulator of *de novo* fatty acid synthesis than fructose in humans warrants investigation.

Second, compared with glucose ingestion, both fructose ingestion and galactose ingestion are known to be much less insulinogenic^(^^4^^,^^5^^)^, which in theory would imply less inhibition on endogenous fat mobilisation, lipolysis and oxidation, and hence a lesser increase in RQ than with glucose. However, in the present study, both these low-glycaemic index sugars (fructose and galactose) elicited greater increases in RQ than glucose ingestion. These findings here, together with the failure of several past studies to show differential fuel partitioning in response to contrasting glycaemic carbohydrates^(^^20^^)^, underscore the fact that there is no direct relationship between glycaemic index and RQ or substrate oxidation. Furthermore, our findings here of a higher postprandial RQ with galactose than with glucose are difficult to reconcile with the results of a recent study involving intermittent sugary drinks showing that galactose intake in substitution for glucose resulted in higher endogenous fat oxidation^(^^9^^)^. It should, however, be pointed out that whereas our study involved young lean men and women, the latter study was performed on middle-aged obese women, and therefore raising the question as to whether obese women may differ from lean subjects in their metabolic responses to galactose. Alternatively, the influence of galactose on energy metabolism might be very different in the presence of glucose or integrated as part of a diet compared with a situation where it is ingested as a drink that only contains the test sugar as energy.

In conclusion, when ingested as a sugary drink, postprandial thermogenesis and utilisation of fat after galactose are not higher than after glucose or fructose. The kinetics of postprandial changes in REE in response to galactose are similar to those for glucose, while the changes in RQ are similar to those for fructose. To what extent postprandial metabolism in response to the low-glycaemia sugar galactose may differ from glucose or fructose when incorporated in meals warrants further investigation.
